# Magnetic Resonance Imaging Investigation of Neuroplasticity After Ischemic Stroke in Tetramethylpyrazine-Treated Rats

**DOI:** 10.3389/fphar.2022.851746

**Published:** 2022-04-26

**Authors:** Xue-Feng Feng, Jian-Feng Lei, Man-Zhong Li, Yu Zhan, Le Yang, Yun Lu, Ming-Cong Li, Yu-Ming Zhuang, Lei Wang, Hui Zhao

**Affiliations:** ^1^ School of Traditional Chinese Medicine, Capital Medical University, Beijing, China; ^2^ Beijing Key Lab of TCM Collateral Disease Theory Research, Beijing, China; ^3^ Medical Imaging Laboratory of Core Facility Center, Capital Medical University, Beijing, China

**Keywords:** tetramethylpyrazine, ischemic stroke, axonal remodeling, synaptic plasticity, white matter reorganization

## Abstract

Ischemic stroke elicits white matter injury typically signed by axonal disintegration and demyelination; thus, the development of white matter reorganization is needed. 2,3,5,6-Tetramethylpyrazine (TMP) is widely used to treat ischemic stroke. This study was aimed to investigate whether TMP could protect the white matter and promote axonal repair after cerebral ischemia. Male Sprague–Dawley rats were subjected to permanent middle cerebral artery occlusion (MCAO) and treated with TMP (10, 20, 40 mg/kg) intraperitoneally for 14 days. The motor function related to gait was evaluated by the gait analysis system. Multiparametric magnetic resonance imaging (MRI) was conducted to noninvasively identify gray-white matter structural integrity, axonal reorganization, and cerebral blood flow (CBF), followed by histological analysis. The expressions of axonal growth-associated protein 43 (GAP-43), synaptophysin (SYN), axonal growth-inhibitory signals, and guidance factors were measured by Western blot. Our results showed TMP reduced infarct volume, relieved gray-white matter damage, promoted axonal remodeling, and restored CBF along the peri-infarct cortex, external capsule, and internal capsule. These MRI findings were confirmed by histopathological data. Moreover, motor function, especially gait impairment, was improved by TMP treatment. Notably, TMP upregulated GAP-43 and SYN and enhanced axonal guidance cues such as Netrin-1/DCC and Slit-2/Robo-1 but downregulated intrinsic growth-inhibitory signals NogoA/NgR/RhoA/ROCK-2. Taken together, our data indicated that TMP facilitated poststroke axonal remodeling and motor functional recovery. Moreover, our findings suggested that TMP restored local CBF, augmented guidance cues, and restrained intrinsic growth-inhibitory signals, all of which might improve the intracerebral microenvironment of ischemic areas and then benefit white matter remodeling.

## Introduction

Ischemic stroke is a grave cause of long-term neurological deficits globally (Chen et al., 2014). Currently, the tissue plasminogen activator (tPA) is the only FDA-approved therapy for ischemic stroke (Hughes et al., 2021), and mechanical thrombectomy has the superiority in treating acute ischemic stroke (Flottmann et al., 2018). Despite stroke mortality being declined with effective thrombolysis in the acute window (4.5 h) after the onset of ischemic stroke, most patients show significant disabilities. An increasing amount of evidence shows that ischemia results in not only mere neuronal loss but also white matter injury signed with axonal disintegration and demyelination, which is correlated with the severity of neurological deficit (Wang et al., 2016). Therefore, neurorestorative treatments for poststroke white matter reorganization and functional recovery are urgently required.

TMP has been extensively used for cardiovascular and cerebrovascular diseases (Chun-sheng et al., 1978; Guo et al., 1983; Zhao et al., 2016). The pharmacokinetics of TMP revealed that the mean residence time (MRT) of TMP administered intravenously in healthy rat blood and brain tissues are 84.0 and 98.2 min respectively, while the MRT of TMP administered intragastrically are 58.9 and 72.9 min, respectively (Wang et al., 2012). Clinical studies demonstrated TMP treatment strengthened survivability, improved neurological functions, and reduced recurrence in stroke patients (Ni et al., 2013).In addition, TMP could reduce cerebral infarct volume, relieve neuronal injury, and protect the blood–brain barrier in stroke models (Tan et al., 2015; Gong et al., 2019). However, whether TMP could protect the structural integrity of white matter, promote axonal remodeling, and expedite long-term functional recovery related to gait is unknown. In this study, we applied MRI technologies combined with histological analysis to explore the potential effectivity of TMP on white matter remodeling, especially axonal reorganization, and evaluated the effect of TMP on gait function by using DigiGait automated gait analysis in the subacute phase of permanent MCAO rats.

Ample evidence has shown that the intrinsic axonal growth-associated signals play a substantial role in axonal regeneration. GAP-43 participates in regulating axonal elongation, and SYN is beneficial to axonal sprouting and synaptogenesis (Chung et al., 2020; Jing et al., 2020). In particular, the coordinated action of attractive and repulsive extracellular axonal guidance molecules, including Netrins and DCC, Slit-2, and Robo-1, is important for neurite growth and guidance (Chen et al., 2020; Cuesta et al., 2020). In addition to neurite growth and guidance, axonal regeneration could be constrained by neurite growth inhibitors. NogoA binding with NgR by initiating the downstream RhoA/ROCK-2 inhibits axonal regeneration and results in growth cone collapse (Wang et al., 2020). Strategies stimulating axonal growth-promoting factors and suppressing growth-inhibiting signals may greatly improve axonal extension and neurological outcomes (Lu et al., 2021). Therefore, in the present study, we examined the expressions of these intrinsic axonal growth-related proteins to gain an insight into the white matter repair mechanisms underlying TMP treatment.

## Materials and Methods

### Animals and Drugs

A total of 76 adult male Sprague–Dawley rats were purchased from Vital River Laboratory Animal Technology Co., Ltd. (Beijing, China) weighing 300–320 g (aged 8 weeks) and were maintained at a specific pathogen-free (SPF) animal research center in Capital Medical University (SYXK [jing] 2018-0003). Animal care and experimental protocols were performed in accordance with the guidelines set by the National Institute of Health Guide for the Care and Use of Laboratory Animals and approved by the Capital Medical University Animal Ethics Committee (Permit Number: AEEI-2018-052).

TMP hydrochloride injection (HPLC >98%) was purchased from Harbin Medisan Pharmaceutical Co., Ltd., (Lot No. 090923A, Harbin, Heilongjiang, China).

### Ischemic Model and Experimental Groups

Focal cerebral ischemia was induced by permanent intraluminal occlusion of the right middle cerebral artery (MCA), according to a previously described method (Laing et al., 1993). The rats were anesthetized with isoflurane (5% for induction and 2% for maintenance) during the surgery. In brief, a right paramidline incision was made to separate the right external carotid artery (ECA) and internal carotid artery (ICA). A small incision was made at the ECA, and a nylon suture (Beijing Sunbio Biotech Co. Ltd., Beijing, China) was inserted into the stump of the ECA. The suture was tightened, and the nylon suture was pushed into the ICA for around 1.8–1.9 cm until a mild sense of resistance was felt to block the origin of the MCA. The rats with successful MCAO showing circling or walking to the contralateral side were included in this experiment (Yu et al., 2013).

Two rats died during MCAO surgery, and four rats that exhibited no obvious neurological symptoms were eliminated from the study. Therefore, 60 MCAO rats were randomly divided into the model group (*n* = 18), TMP 40 mg/kg group (*n* = 14), TMP 20 mg/kg (*n* = 14) group, and TMP 10 mg/kg group (*n* = 14) by experimenters blinded to the treatment conditions. There were no group differences in neurological deficit scores and body weight before treatment. Another 10 sham-operated rats were grouped into the sham group. TMP dissolved in saline was intraperitoneally injected to rats 4 h after MCAO and once daily for 14 days. The rats in the sham and model groups were injected with the same volume of saline (1 ml/kg/day). The rats from the model group (*n* = 12), TMP 40 mg/kg group (*n* = 12), TMP 20 mg/kg group (*n* = 11), TMP 10 mg/kg group (*n* = 11), and all the sham-operated rats were able to survive.

### Rat Gait Analysis

Gait assessment was carried out on day 14 after MCAO using the DigiGait Imaging and Analysis 15.0 system (Mouse Specifics, Inc., Boston, United States) (Hampton et al., 2004). In brief, rats (*n* = 10 per group) were trained to walk as the speed was gradually increased to 15 cm/s. A high-speed video camera mounted below captured four paws and their positions relative to the belt, and qualified videos contained at least three successive footprints (Feng et al., 2019).

Gait parameters were summarized as follows: steps (the number of steps in a stride cycle), cadence (the number of steps taken per second) (Parkkinen et al., 2013), hindlimb shared stance time (the time in contact with the belt with hindlimbs), stride length (the distance between two successive initial postures during the maximal contact), paw area (the maximal paw area in contact with the belt), and ataxia coefficient (Caballero-Garrido et al., 2017). Gait detection and data analysis were conducted by two experimenters blinded to the group assignment.

### MRI Acquisition and Analysis

MRI measurements were performed with a 7.0 T MRI scanner (Bruker, PharmaScan, Germany) on the 15^th^ day after TMP intervention (*n* = 6 per group). The rats were anesthetized (5% isoflurane for induction and 2% isoflurane for maintenance) with an anesthesia system (JD Medical Dist. Co. Inc., United States). MRI images were reconstructed by Paravision version 5.1 software (Bruker, PharmaScan, Germany).

T2-weighted imaging (T2WI) was conducted with a fast spin-echo pulse sequence with the following parameters: repetition time (TR) = 4,400 ms, echo time (TE) = 45 ms, field of view (FOV) = 3 × 3 cm^2^ ,and matrix size (MS) = 256 × 256 (Li M Z et al., 2019). Infarct regions were defined by the areas with hyperintensity on T2 images (Chan et al., 2009). The infarct volume was calculated as the summation of infarct areas by the slice thickness (0.7 mm) by ImageJ software (Liu et al., 2011). Similarly, the volumes of bilateral hemispheres and ventricles were calculated. The ipsilateral residual tissue volume was equal to subtracting the infarct and ventricular volumes from the hemisphere volume (Li M et al., 2018).

T2 relaxometry mapping was used to analyze tissue lesions with a multislice multiecho sequence with the following parameters: TR = 2,500 ms, TEs from 11 to 176 ms, FOV = 3.3 × 3.3 cm^2^, and MS = 256 × 256 (Zhang et al., 2016). Regions of interest (ROIs) were manually delineated in the bilateral peri-infarct cortex, external capsule, internal capsule, motor cortex, and somatosensory cortex, following a rat atlas (Schober, 1986). T2 values of ROIs were obtained on coronal T2 relaxometry maps by Paravision version 5.1 software. The relative T2 (rT2) was calculated as the ipsilateral T2 value relative to the contralateral T2 value.

Diffusion tensor imaging (DTI) was conducted to detect the microstructural changes with an axial single-shot spin echo-planar imaging sequence with the following parameters: TR/TE = 6,300/25 ms, 30 diffusion encoding directions, and b values = 0, 1,000 s/mm^2^ (Zhang et al., 2016). The images of fractional anisotropy (FA), apparent diffusion coefficient (ADC), axial diffusivity (AD), and radial diffusivity (RD) were reconstructed with Paravision version 5.1 software. ROIs were delineated in the bilateral peri-infarct cortex, external capsule, and internal capsule on DTI parametric maps to obtain DTI values (Schober, 1986). Diffusion tensor tractography (DTT) was reconstructed with DSI studio and Diffusion Toolkit software to determine the orientation and integrity of nerve fibers (Liu et al., 2011). The mean fiber length and density of the external capsule and internal capsule were measured (Li M. Z et al., 2018). Data were presented as the ratio of ipsilateral values relative to contralateral values (Guo et al., 2011).

Arterial spin labeling (ASL) was performed to quantify the CBF with an echo-planar imaging fluid-attenuated inversion recovery sequence with the following parameters: TR/TE = 18,000/25 ms, FOV = 3.0 × 3.0 cm^2^, matrix size = 128 × 128, and number of excitations = 1. ASL raw data and CBF maps were obtained by Paravision version 5.1 software. The CBF values (mL/100 g/min) of the bilateral peri-infarct cortex, external capsule, and internal capsule were acquired based on our previous method (Zhang et al., 2016; Zhang et al., 2019). The relative CBF (rCBF) was the ratio of the ipsilateral CBF to the contralateral CBF.

### Tissue Examination

After MRI scanning, rats were anesthetized for histologic evaluation and ultrastructural detection. The brains of rats were processed as previously described (Zhan et al., 2020). Hematoxylin and eosin (HE) staining was performed to identify the pathological injury of brain tissues (*n* = 4 per group). The number of nerve cells was measured from three non-overlapping microscopic regions randomly selected in the peri-infarct cortex, according to the previously described method (Li M Z et al., 2019). Data were presented by the average number of cells per mm^2^.

Luxol fast blue (LFB) staining was carried out to observe myelinated axon damage (Patro et al., 2019) (*n* = 4 per group). Three microscopic fields were randomly sampled from the bilateral external capsule and internal capsule. The integrated optical density (IOD) in LFB staining was analyzed with the NIS-Elements Basic Research Image Collection Analysis system (Nikon, Japan). Data were expressed as the ratio of the ipsilateral IOD to the contralateral IOD (Li M Z et al., 2019).

### Transmission Electron Microscope Analysis

The ultrastructural changes in axons and synapses in the peri-ischemic cortex were examined with H7700TEM (Hitachi, Tokyo, Japan) (*n* = 2 per group). In order to evaluate the documentation and arrangement of axonal remyelination, an average of 43 images of axons were captured from each group, and G-ratio (axonal diameter/total fiber diameter) was analyzed (Ramadan et al., 2017). In addition, the synaptic plasticity was analyzed with an average of 20 synapses per group. The number of vesicles in presynaptic membranes was counted, and ultrastructural synaptic junctions including the presynaptic membrane length, synaptic cleft width, postsynaptic density (PSD) thickness, and postsynaptic membrane curvature were analyzed, as described previously (Xu et al., 2009).

Furthermore, the damage degree of mitochondria in axons and synapses was scored, according to the evaluation standard: grade 0, normal structure with intact mitochondrial matrix granules; grade 1, absent mitochondrial matrix granules; grade 2, swollen mitochondria and transparent matrix; grade 3, the disintegrating structure of mitochondrial cristae; and grade 4, destructive bilayer membranes of mitochondria (Hou et al., 2016).

### Western Blot Analysis

The rats (*n* = 4 per group) without undergoing MRI experiments were deeply anesthetized. The perilesional cortex was separated, and protein levels were determined by Western blotting, as previously described (Zhan et al., 2020). Proteins were transferred onto polyvinylidene difluoride membranes, followed by blocking them with 5% nonfat milk for 2 h and subsequently incubating membranes at 4°C overnight with primary antibodies: anti-GAP-43 (1:40000; Epitomics, #2259-1), SYN (1:320000; Epitomics, #1870-1), Netrin-1 (1:2,000; Abcam, ab126729), DCC (1:1,000; Abcam, ab125280), Slit-2 (1:10,000; Abcam, ab134166), Robo-1 (1:1,000; Abcam, ab7279), NogoA (1:20,000; Abcam, ab62024), NgR (1:40,000; Abcam, ab62024), RhoA (1:20,000; Cell signaling, 2117s), ROCK-2 (1:50,000; Abcam, ab125025), and GAPDH (1:1,60,000; GeneTex, GTX627408). After washing, membranes were incubated with secondary anti-rabbit (1:20,000; Applygen Technologies Inc., C1309) or anti-mouse (1:20,000; NeoBioscience, cat. ANM 02-1, Lot. 0912) IgG (H + L)-HRP for 1 h at room temperature. Immunoreactive protein bands were detected by using the SuperECL Plus kit (Applygen, China, cat. No. P1050) and chemiluminescent imager (VILBER, United States). The intensities of target proteins were quantified by ImageJ software.

### Statistical Analysis

Data were presented as mean ± standard error of the mean (SEM). Statistical analysis was performed using the SPSS 26.0 software (SPSS Inc., United States). Data were analyzed with a one-way analysis of variance (ANOVA), followed by Bonferroni’s *post hoc* test. Pearson linear regression was conducted to analyze the correlation between rCBF and DTI metrics. The statistical significance was defined as *p* < 0.05.

## Results

### TMP Alleviated Cerebral Infarction

**FIGURE 1:**
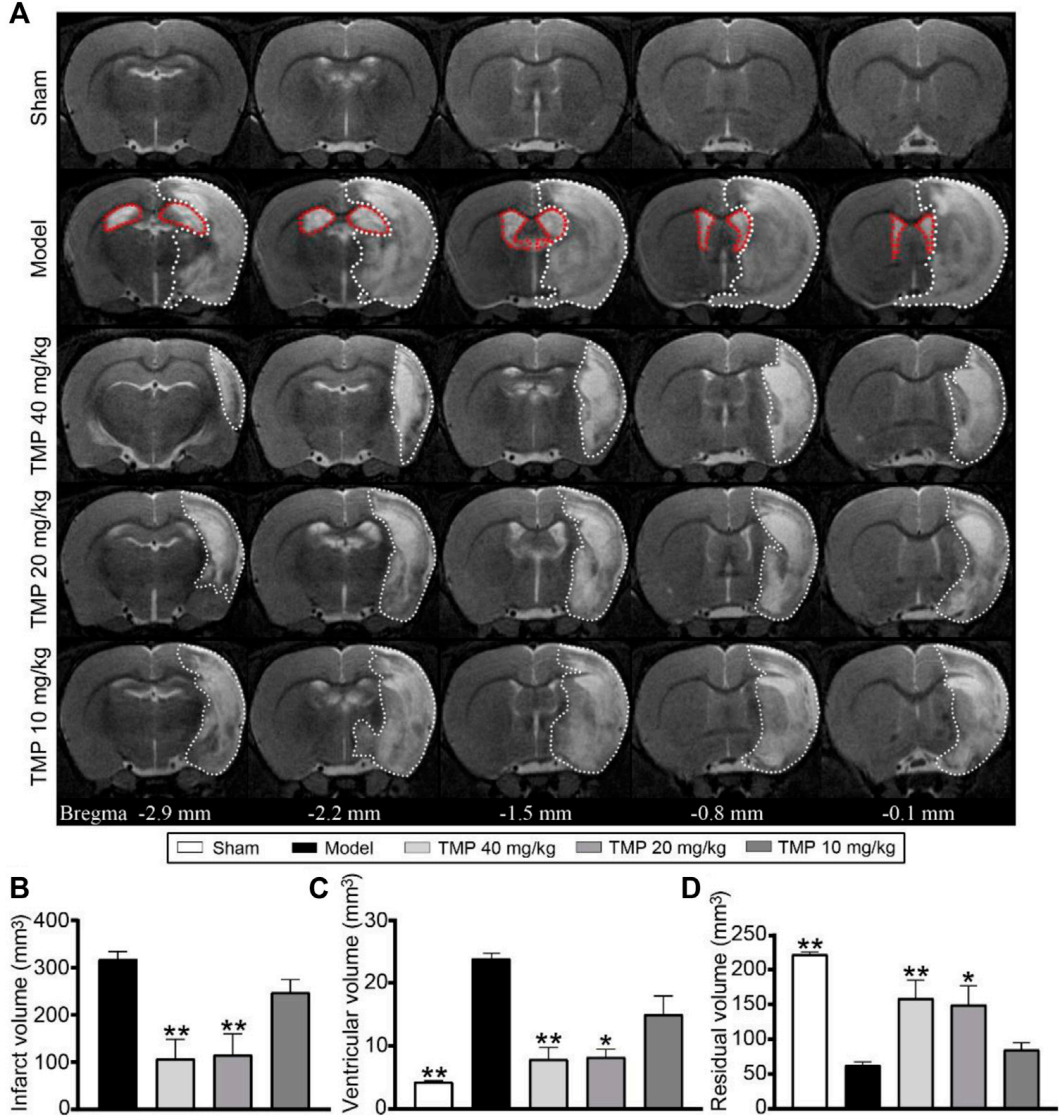
Effect of TMP on cerebral infarction in MCAO rats. **(A)** Typical axial T2 images of each group. The infarct areas are represented with white dotted lines. The ventricles are represented with red dotted lines in the model group (*n* = 6). Quantitative analysis of the **(B)** infarct volume, **(C)** ventricular volume, and **(D)** residual volume. *p < 0.05 and **p < 0.01 vs. Model group.

T2WI revealed a hyperintense signal in the MCA territory, indicating tissue infarction (Figure 1A). TMP (20, 40 mg/kg) treatment obviously decreased the infarct volume compared with the model group (*p* < 0.01) (Figure 1B). Notably, in comparison with sham rats, the bilateral ventricles were severely enlarged, and the ipsilateral residual volume was decreased after ischemia for 15 days (*p* < 0.01). TMP (20, 40 mg/kg) effectively relieved the ventricular dilatation and preserved the residual tissues in comparison with model rats (*p* < 0.05 or *p* < 0.01) (Figures 1C,D).

### TMP Relieved Cerebral Tissue Injury and Protected the Myelinated Axons

**FIGURE 2:**
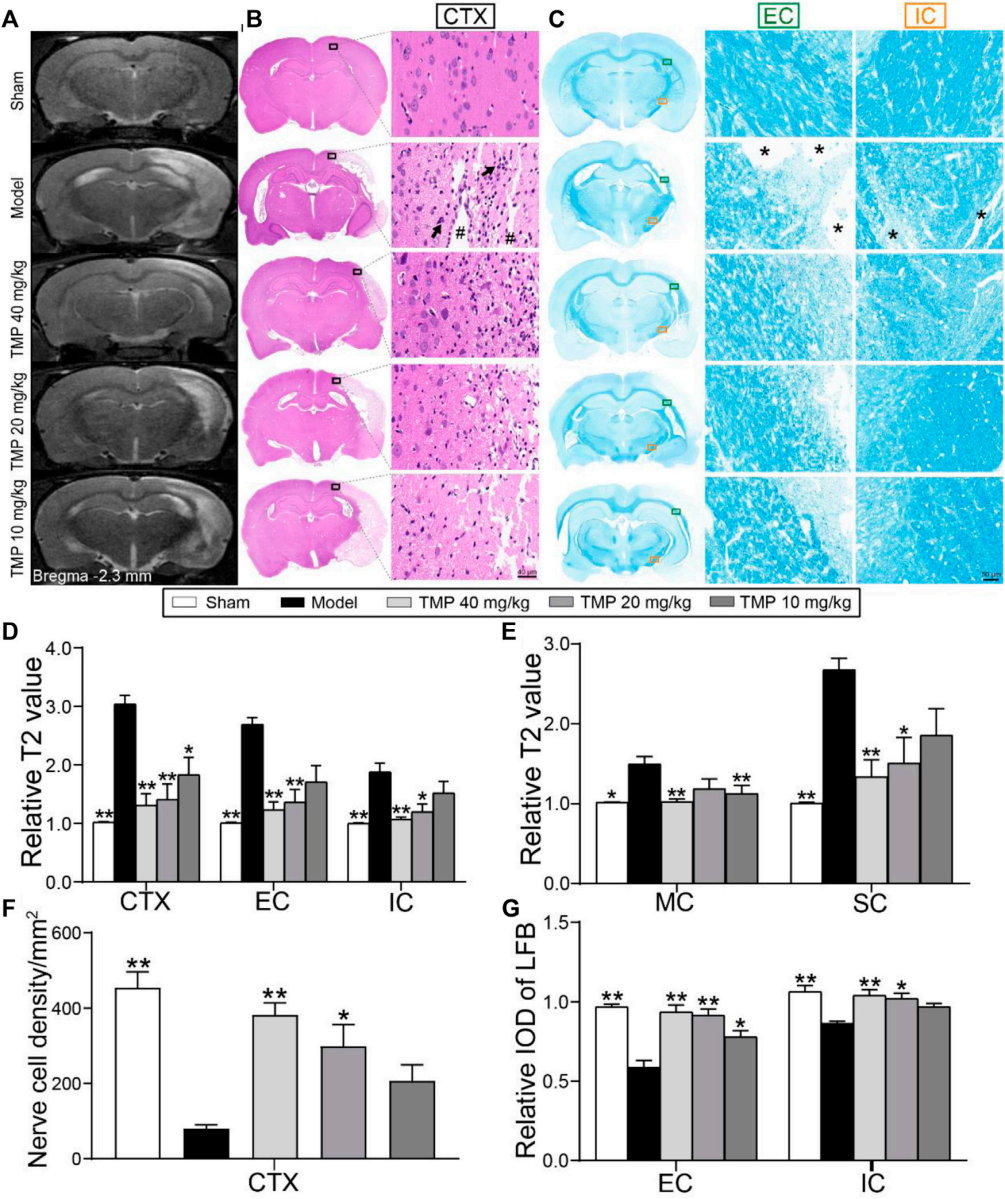
Effect of TMP on cerebral tissue injury in MCAO rats. **(A)** Typical T2 relaxometry mapping of each group (*n* = 6). **(B)** Typical HE staining photographs showed the degeneration and necrosis of the nerve cells (black arrows) in the peri-infarct cortex (CTX, black boxes) and destructive tissues (#) indicated in the model group (*n* = 4). **(C)** Typical LFB staining photographs showed myelin sheath of the external capsule (EC, green boxes) and internal capsule (IC, orange boxes). Remarkable cavitation areas with myelin sheath loss (*) in nerve fibers were indicated in the model group (n = 4). Quantitative analysis of relative T2 values of **(D)** CTX, EC, and IC, **(E)** motor cortex (MC), and somatosensory cortex (SC). Quantitative analysis of **(F)** the nerve cell density of CTX and **(G)** relative LFB-IOD of EC and IC. *p < 0.05 and **p < 0.01 vs. Model group.

T2 relaxometry mapping was conducted to determine structural changes in the gray and white matter (Figure 2A). Quantitative analysis showed higher rT2 values were detected in the ipsilateral gray matter (the peri-infarct cortex) and white matter (the external capsule and internal capsule) areas of model rats compared with sham rats (*p* < 0.01). In contrast, TMP (20, 40 mg/kg)-treated rats showed lower rT2 in the peri-infarct cortex, somatosensory cortex, external capsule, and internal capsule (*p* < 0.05 or *p* < 0.01), and TMP (10 mg/kg) decreased rT2 of the peri-infarct and motor cortex as compared with model rats (*p* < 0.05 or *p* < 0.01). TMP (40 mg/kg) additionally decreased rT2 of the motor cortex compared to the model group (*p* < 0.01) (Figures 2D,E).

Furthermore, HE staining (Figure 2B) showed that the nerve cell density of the peri-infarct cortex was sharply decreased when compared with the sham group (*p* < 0.01), whereas a significantly higher number of nerve cells was found in TMP (20, 40 mg/kg) treatment groups than in the model group (*p* < 0.05 or *p* < 0.01) (Figure 2F).

LFB staining was performed to examine the alternations of axons and myelin sheath (Figure 2C). In comparison with the sham group, the relative LFB-IODs of the external capsule and internal capsule in model rats were sharply decreased (*p* < 0.01). The relative IODs of the external capsule and internal capsule were increased in TMP (20, 40 mg/kg)-treated rats, and TMP (10 mg/kg) also enhanced the relative LFB-IOD of the internal capsule (*p* < 0.05 or *p* < 0.01) (Figure 2G). In conclusion, TMP plays a remarkable role in protecting nerve cells in gray matter and myelinated axons in white matter after ischemia.

### TMP Ameliorated the Damage of the Axonal Microstructure

**FIGURE 3:**
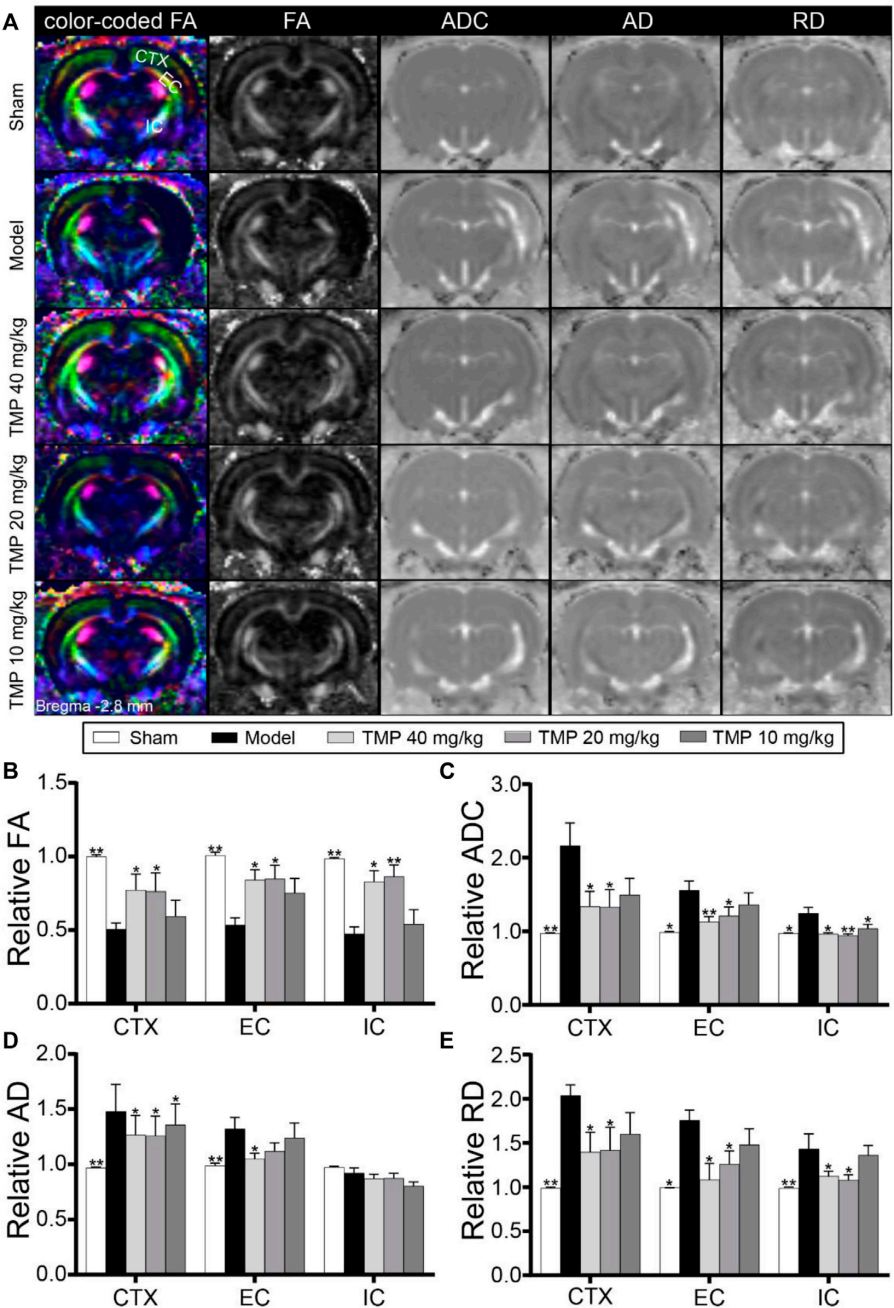
Effect of TMP on the axonal microstructure in MCAO rats. **(A)** Typical axial color-coded FA, FA, ADC, AD, and RD images of each group. ROIs of the peri-infarct cortex (CTX), external capsule (EC), and internal capsule (IC) were identified on the color-coded FA images in the sham group (*n* = 6). **(B–E)** Quantitative analysis of the relative FA, ADC, AD, and RD of CTX, EC, and IC, respectively. *p < 0.05 and **p < 0.01 vs. Model group.

DTI was utilized to evaluate the microstructural changes in axons (Figure 3A). First, the DTI-derived parameter FA characterizes the alterations of the axonal microstructure. Quantitative data demonstrated that rFA was sharply decreased in the model perilesional cortex, external capsule, and internal capsule compared to the sham group (*p* < 0.01), while it was reversed by TMP (20, 40 mg/kg) (*p* < 0.05 or *p* < 0.01) (Figure 3B). In addition, rADC is employed to detect cellular damage. As shown in model rats, the increased rADC was detected in the peri-infarct cortex, external capsule, and internal capsule. In contrast, TMP (20, 40 mg/kg) decreased rADC of the peri-infarct cortex and external capsule compared with the model rats (*p* < 0.05 or *p* < 0.01), and the reduced rADC was also detected in the internal capsule of TMP (10 mg/kg) group rats (*p* < 0.05) (Figure 3C).

In particular, rAD and rRD were respectively used to analyze the alterations of axons and myelin sheath. DTI results showed elevated rAD and rRD in the peri-infarct cortex, external capsule, and internal capsule of model rats compared with sham rats (*p* < 0.05 or *p* < 0.01). After the treatment with TMP (10, 20, and 40 mg/kg), the rAD of the peri-infarct cortex was significantly reduced as compared to model rats (*p* < 0.05). Moreover, TMP (40 mg/kg) also decreased rAD of the ipsilateral external capsule (Figure 3D). Moreover, TMP (20, 40 mg/kg) remarkably decreased rRD in the peri-infarct cortex, external capsule, and internal capsule in comparison with the model group (*p* < 0.05 or *p* < 0.01). These results suggest that TMP could ameliorate the damage to the axonal microstructure in ischemic rats.

### TMP Facilitated Axonal Restoration

**FIGURE 4:**
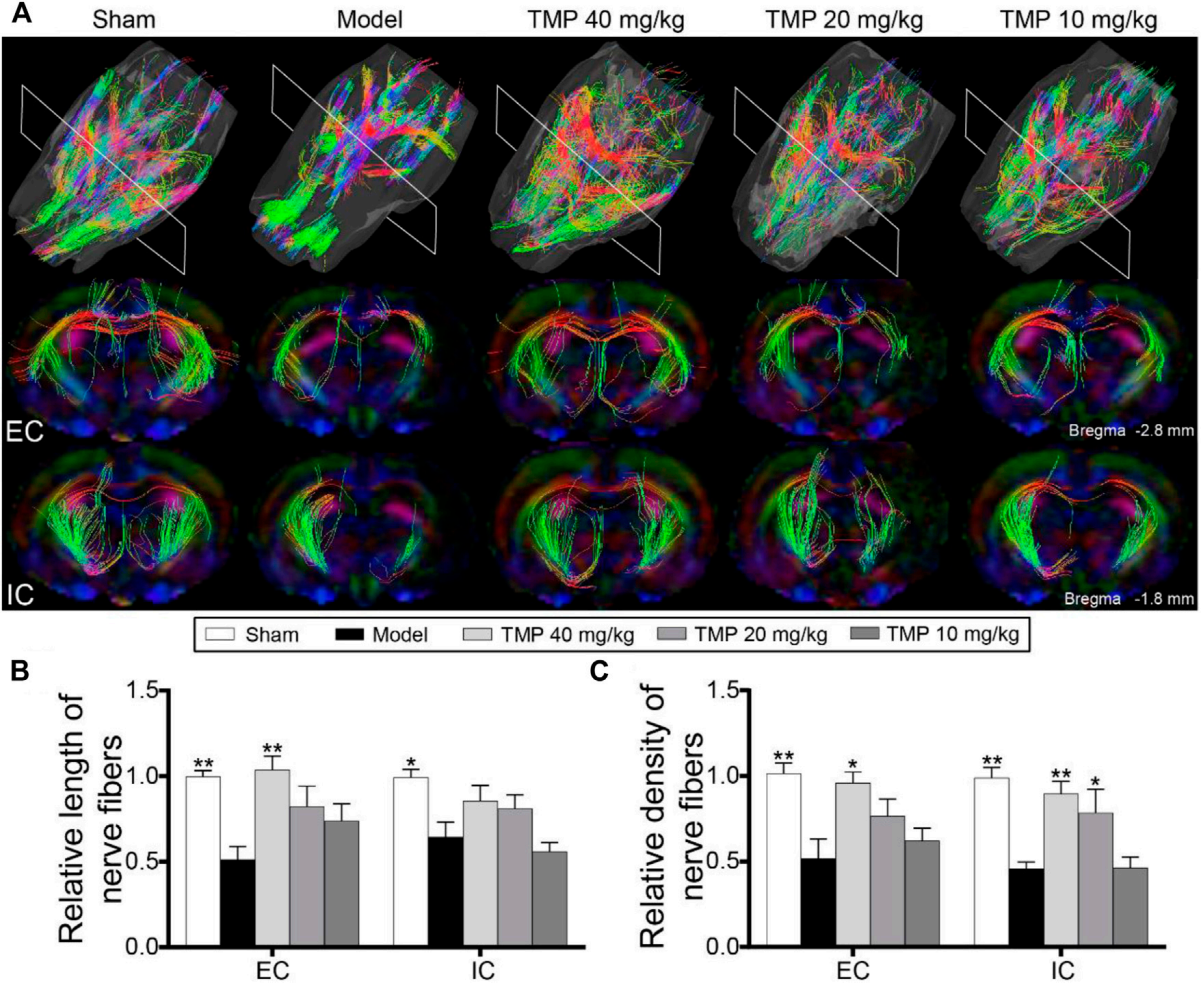
Effect of TMP on axonal restoration in MCAO rats. **(A)** Typical 3D reconstruction DTT images of the whole brain and anatomical pathways of the external capsule (EC) and internal capsule (IC) of each group (*n* = 6). Quantitation of the **(B)** relative length and **(C)** relative density of nerve fibers in EC and IC. *p < 0.05 and **p < 0.01 vs. Model group.

DTT was conducted to demonstrate the integrity and connectivity of nerve fibers (Figure 4A). The model group rats showed decreased relative fiber length and density in the external capsule and internal capsule compared to sham rats (*p* < 0.01). After treatment with TMP (40 mg/kg), the relative fiber length and density of the external capsule were remarkably increased, and the relative density of internal capsule fibers was also increased in comparison with model rats (*p* < 0.05 or *p* < 0.01). Additionally, TMP (20 mg/kg) elevated the relative density of internal capsule fibers in comparison with model rats (*p* < 0.05 or *p* < 0.01) (Figures 4B,C).

### TMP Improved the Cerebral Perfusion

**FIGURE 5:**
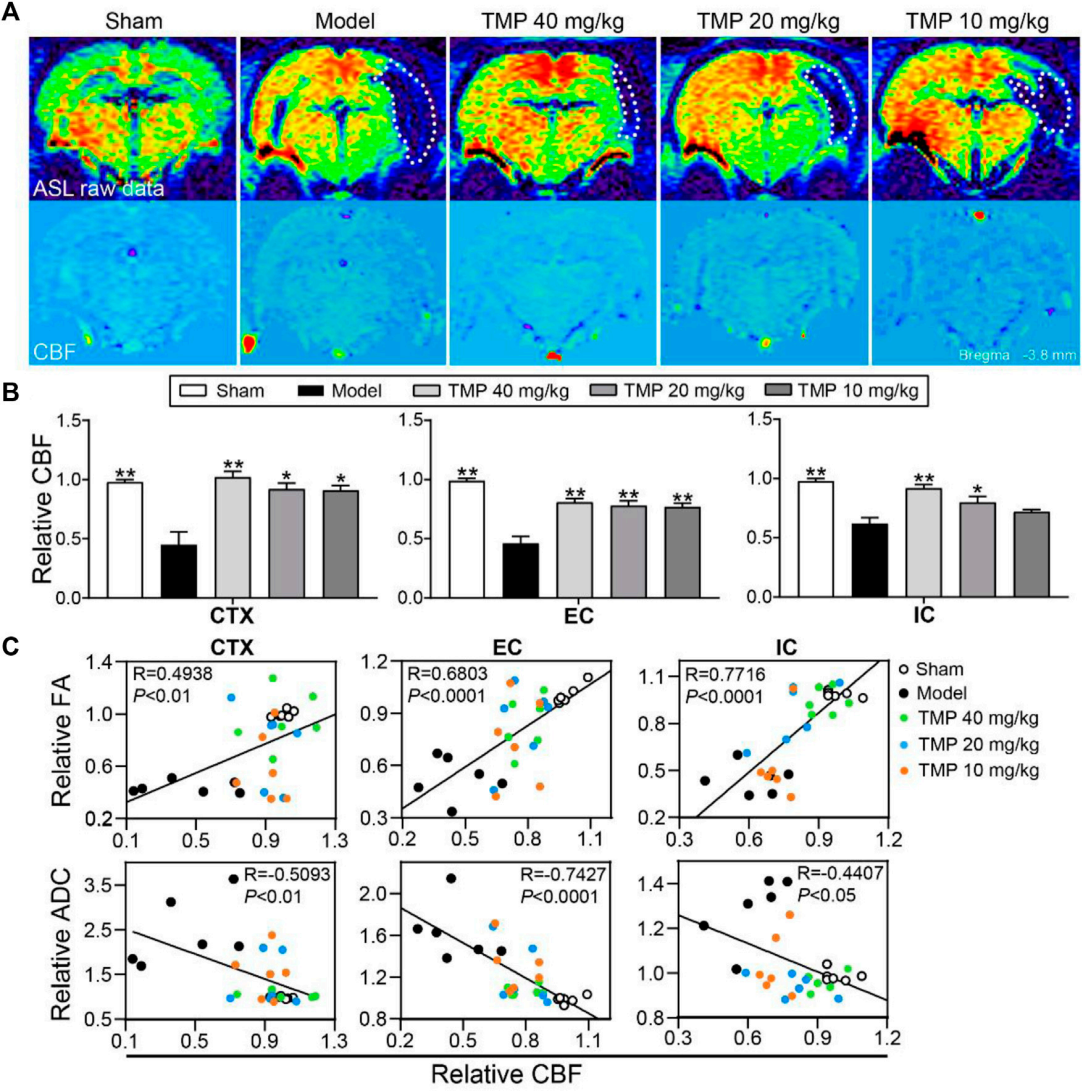
Effect of TMP on cerebral perfusion in MCAO rats. **(A)** Typical ASL raw data and CBF images of each group. The ischemic areas are represented with white dotted lines (*n* = 6). **(B)** Quantitation of relative CBF (rCBF) of the peri-infarct cortex (CTX), external capsule (EC), and internal capsule (IC). **(C)** Correlations between DTI metrics (rFA and rADC) and rCBF of CTX, EC, and IC. *p < 0.05 and **p < 0.01 vs. Model group.

Cerebral perfusion was quantitatively evaluated with ASL (Figure 5A). *Post hoc* comparisons revealed the rCBF of the peri-infarct cortex, external capsule, and internal capsule in model rats was significantly decreased in comparison to the sham group (*p* < 0.01). TMP treatment (20, 40 mg/kg) significantly increased the rCBF of the peri-infarct cortex, external capsule, and internal capsule (*p* < 0.05 or *p* < 0.01), and TMP (10 mg/kg) also increased the rCBF of the peri-infarct cortex and external capsule compared to model rats (*p* < 0.05 or *p* < 0.01) (Figure 5B).

Furthermore, Pearson linear regression analysis showed rFA was significantly in positive correlation with rCBF in the peri-infarct cortex (R = 0.4938 and *p* < 0.01), external capsule (R = 0.6803 and *p* < 0.0001), and internal capsule (R = 0.7716 and *p* < 0.0001). Meanwhile, rADC was strongly in negative correlation with rCBF in the peri-infarct cortex (R = -0.5093 and *p* < 0.01), external capsule (R = -0.7427 and *p* < 0.0001), and internal capsule (R = −0.4407 and *p* < 0.05), suggesting the improvement of the rCBF might contribute to restore the axonal microstructure after ischemia (Figure 5C).

### TMP Elevated GAP-43 and SYN Expressions and Decreased Damage to Axonal and Synaptic Microstructures

**FIGURE 6:**
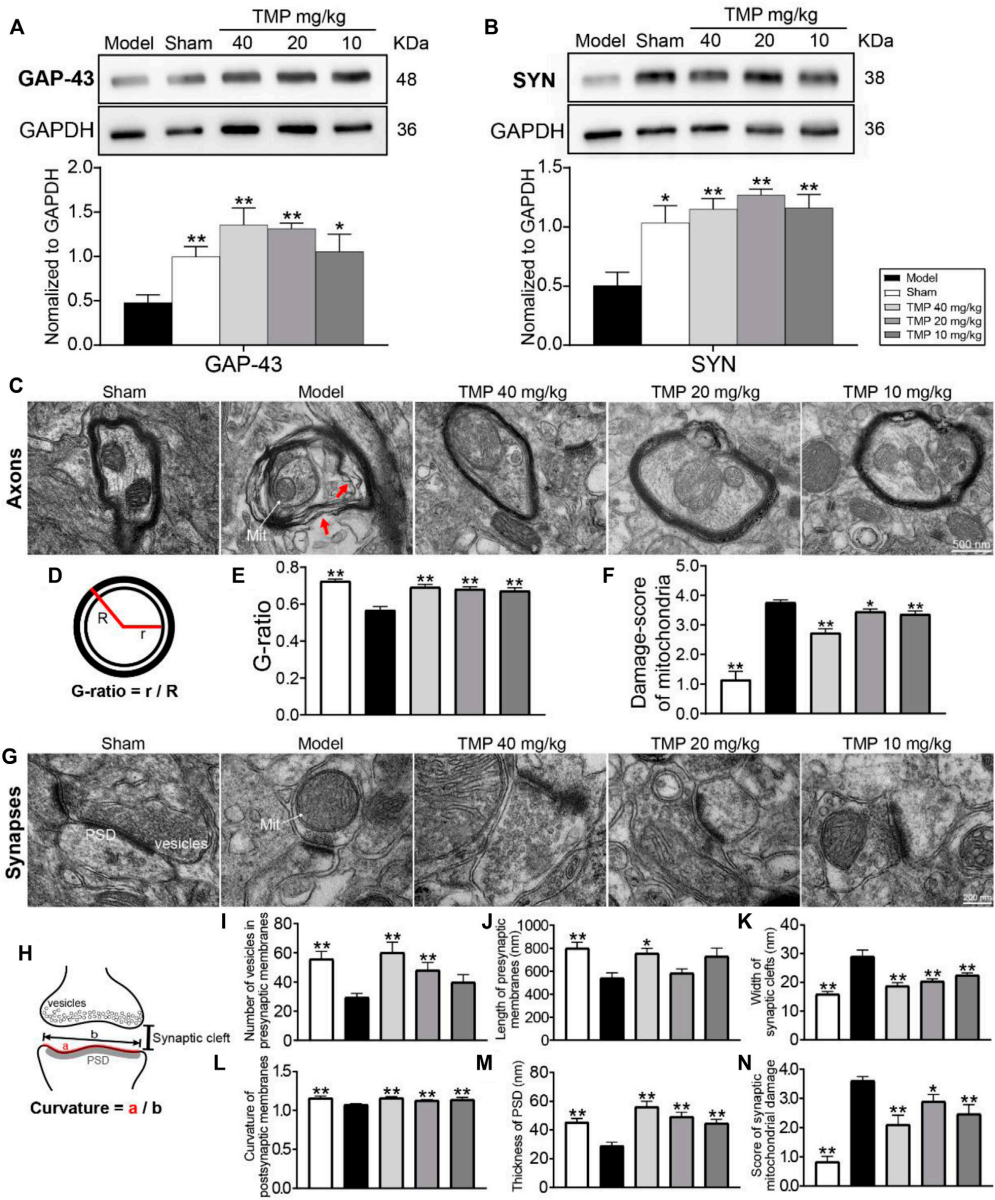
Effects of TMP on GAP-43 and SYN expressions and axonal and synaptic microstructural damage in MCAO rats. Typical Western blot images and analysis for **(A)** GAP-43 and **(B)** SYN (*n* = 4). The protein levels were quantified with GAPDH as the loading control. **(C)** Representative TEM images of myelinated axons in each group, and the axons separating from myelin sheath (red arrows) and abnormal mitochondria (Mit) were indicated in the model group (*n* = 2). **(D)** G-ratio indicated the axonal diameter (r)/total fiber diameter (R). The quantitation of **(E)** G-ratio and **(F)** damage score of mitochondria in axons. **(G)** Representative TEM images of synapses in each group (*n* = 2). **(H)** Vesicles in presynaptic membranes, synaptic cleft, PSD, and postsynaptic membrane curvature are shown on the schematic diagram. The quantitation of **(I)** vesicles in presynaptic membranes, **(J)** presynaptic membrane length, **(K)** synaptic cleft width, **(L)** postsynaptic membrane curvature, **(M)** PSD thickness, and **(N)** damage score of mitochondria in synapses. *p < 0.05 and **p < 0.01 vs. Model group.

To test whether TMP treatment of stroke induces axonal and synaptic plasticity, GAP-43 (a marker for axon growth) and SYN (a marker for synaptogenesis) were examined. Western blot revealed that GAP-43 and SYN in the model perilesional cortex were significantly decreased compared with the sham group (*p* < 0.05 or *p* < 0.01). After TMP (10, 20, and 40 mg/kg) treatment, GAP-43 and SYN were significantly elevated in comparison with the model cortex (*p* < 0.05 or *p* < 0.01) (Figures 6A,B).

Notably, electron micrographs showed the ultrastructural alterations of myelinated axons with the swollen myelin lamina after MCAO (Figure 6C). Moreover, the measurement of the G-ratio was significantly declined in the model group as compared with the sham group (*p* < 0.01). TMP (10, 20, and 40 mg/kg) dramatically increased the G-ratio in comparison with model rats (*p* < 0.01), suggesting axonal remyelination after TMP intervention (Figure 6C).

In addition, the synaptic ultrastructural analysis showed MCAO decreased the number of vesicles in presynaptic membranes, induced structural changes in synaptic junctions including the decreased presynaptic membrane length, PSD thickness, and postsynaptic membrane curvature, and increased the synaptic cleft width. The synaptic parameters were altered after TMP treatment. TMP (10, 20, and 40 mg/kg) increased PSD thickness and postsynaptic membrane curvature and decreased synaptic cleft width in comparison with model rats (*p* < 0.01). In particular, TMP (20, 40 mg/kg) increased the number of presynaptic vesicles (*p* < 0.01), and TMP (40 mg/kg) also extended the presynaptic membrane length (*p* < 0.05) (Figures 6I–M-M).

Moreover, quantitation showed that mitochondrial damage scores in axons and synapses were increased in the model group compared to the sham group (*p* < 0.01). TMP (10, 20, and 40 mg/kg) restored the mitochondrial injury in the perilesional cortex (*p* < 0.05 or *p* < 0.01) (Figures 6F,N).

### TMP Regulated Axonal Guidance Signals and Inhibited Axonal Growth-Inhibitory Signals

**FIGURE 7:**
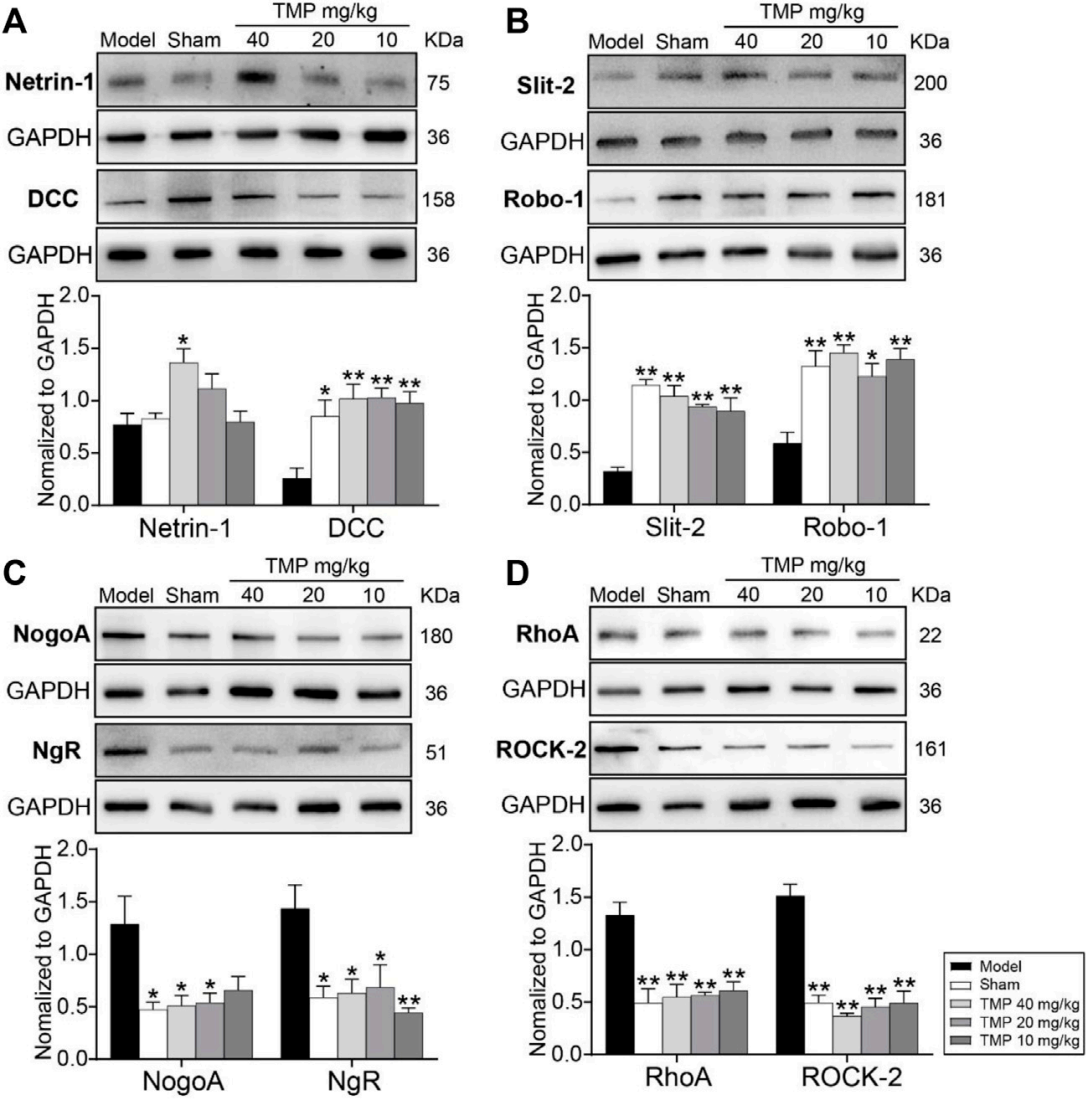
Effects of TMP on the expressions of axonal guidance and growth-inhibitory signals in MCAO rats. Typical Western blot images and analysis for **(A)** Netrin-1/DCC, **(B)** Slit-2/Robo-1, **(C)** NogoA/NgR, and **(D)** RhoA/ROCK-2 (*n* = 4). The protein levels were quantified with GAPDH as the loading control. *p < 0.05 and **p < 0.01 vs. Model group.

Western blot results showed that axonal guidance factors DCC, Slit-2, and Robo-1 were notably downregulated in the periischemic cortex of model rats compared to sham rats (*p* < 0.05 or *p* < 0.01), while TMP (10, 20, and 40 mg/kg) significantly upregulated DCC, Slit-2, and Robo-1 expressions compared to the model group (*p* < 0.05 or *p* < 0.01). In addition, TMP (40 mg/kg) also increased the expression of Netrin-1 when compared to model rats (*p* < 0.05) (Figures 7A,B).

In particular, the axonal growth inhibitors NogoA/NgR and RhoA/ROCK-2 were distinctly upregulated in model rats compared with the sham group (*p* < 0.05 or *p* < 0.01). In comparison with the model group, TMP (20, 40 mg/kg) treatment downregulated NogoA, NgR, RhoA, and ROCK-2, and TMP (10 mg/kg) also suppressed NgR, RhoA, and ROCK-2 levels in the peri-ischemic cortex (Figures 7C,D). To sum up, TMP improves axonal remodeling by regulating axonal guidance and growth-inhibitory signals.

### TMP Improved the Gait Function

**FIGURE 8:**
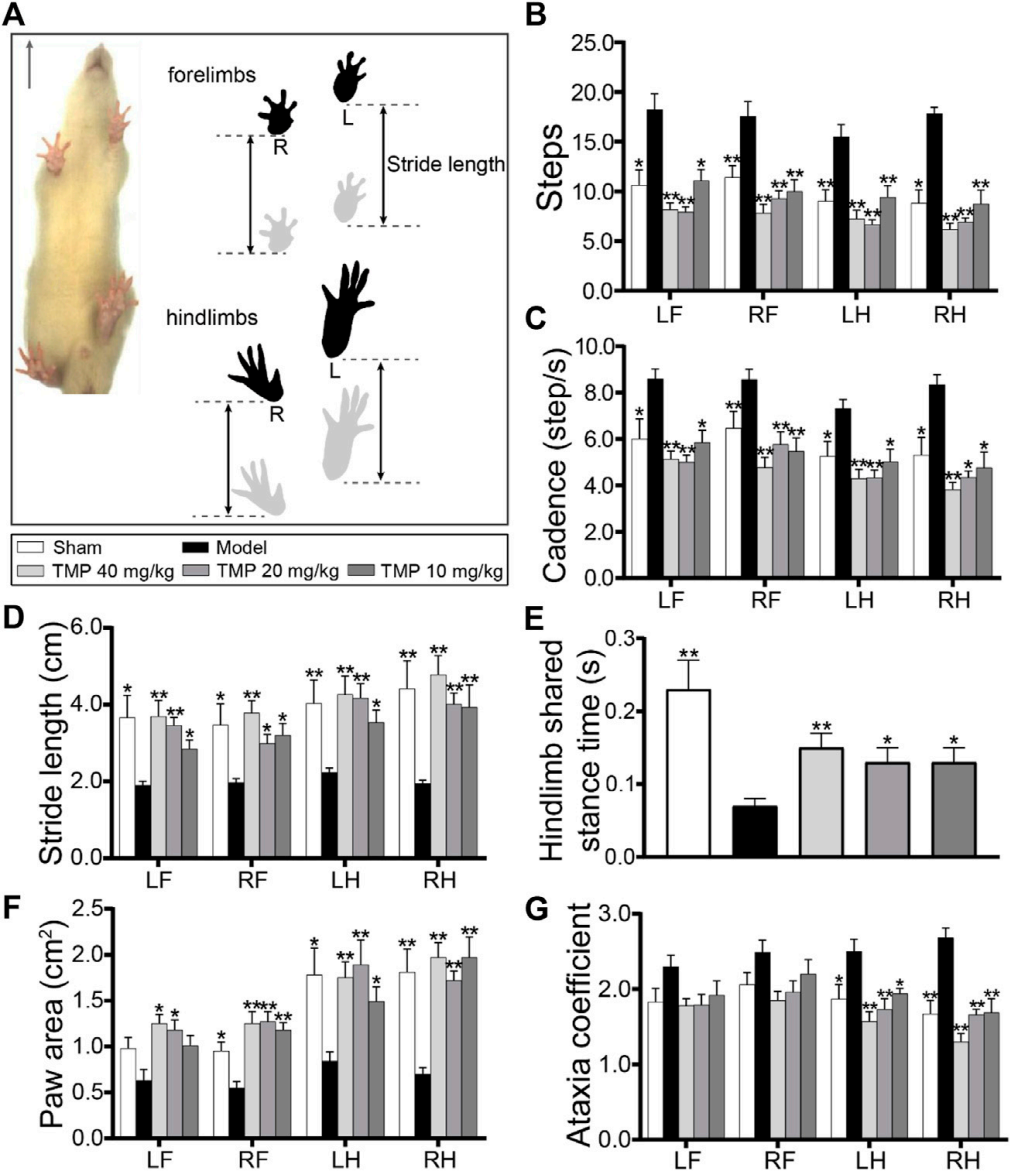
Effects of TMP on gait functions in MCAO rats. **(A)** Schematic diagram exhibited a rat subjected to a gait test (*n* = 10). The locations of the four paws were painted. Stride length indicated the distance between two successive initial postures during the maximal contact. Quantitative analysis of **(B)** steps, **(C)** cadence, **(D)** stride length, **(E)** hindlimb shared stance time, **(F)** paw area, and **(G)** ataxia coefficient in rats of each group. LF, left forelimb; RF, right forelimb; LH, left hindlimb; RH, right hindlimb. *p < 0.05 and **p < 0.01 vs. Model group.

The gait impairment and functional recovery after TMP treatment were evaluated by the DigiGait-automated gait test (Figure 8A). The model rats displayed the significantly increased steps and cadence compared with sham rats (*p* < 0.05 or *p* < 0.01). After TMP (10, 20, and 40 mg/kg) treatment, steps and cadence were decreased in comparison with model rats (*p* < 0.05 or *p* < 0.01) (Figures 8B,C). The stride length of the four limbs and hindlimb shared stance time were shortened in model rats compared to sham rats (*p* < 0.05 or *p* < 0.01), which were both reversed by TMP (10, 20, and 40 mg/kg) (*p* < 0.05 or *p* < 0.01) (Figures 8D,E).

The paw area of the left hindlimb and right limbs in model rats was smaller than that in sham rats (*p* < 0.05 or *p* < 0.01), which was increased in TMP (10, 20, and 40 mg/kg) groups, and TMP (20, 40 mg/kg) also increased the paw area of the right forelimbs in comparison with the model group (*p* < 0.05) (Figure 8F).

Notably, the model rats showed an increased ataxia coefficient of the hindlimbs compared with the sham group, representing the impairment in the interlimb coordination (*p* < 0.05 or *p* < 0.01), whereas it was decreased by TMP (10, 20, and 40 mg/kg) compared with model rats (*p* < 0.05 or *p* < 0.01) (Figure 8G). These data elucidate that TMP could alleviate gait impairment and improve the limb locomotor function after ischemic stroke.

## Discussion

On the basis of the multiparametric MRI, the present study revealed that TMP had beneficial effects on protecting both gray and white matter, especially promoting axonal remodeling after ischemic stroke, which was coupled with improving cerebral perfusion. With the information obtained from the DigiGait-automated analysis, we demonstrated that TMP facilitated gait function recovery. Meanwhile, intrinsic axonal guidance cues and growth-inhibitory signals would be participated in the process of TMP promoting axonal reorganization. Our research would offer an ideal therapeutic approach to promote post-stroke brain repair.

MRI is an extremely reliable modality to noninvasively reveal tissue damages (Saar and Koretsky, 2019). In the present study, T2WI images exhibited TMP could reduce infarct volumes and protect residual tissues of ischemic brains, and T2 relaxometry mapping displayed decreased T2 values located in the peri-infarct cortex, external capsule, and internal capsule, following TMP treatment. In corresponding areas, histopathological analysis confirmed TMP preserved nerve cells in the peri-infarct cortex and increased LFB positive myelinated nerve fibers in the external capsule and internal capsule which are the integral white matters. Therefore, our data indicated TMP not only relieves gray matter injury but also protects white matter structures.

We noninvasively detected the microstructure and integrity of gray and white matter by DTI (Jung et al., 2017). The decreased rFA but increased rADC, rAD, and rRD predominantly appeared in the peri-infarct cortex, external capsule, and internal capsule after stroke. FA as a common parameter of DTI reflects the density and integrity of the axonal microstructure, while the reduced FA pixels independently represent demyelination and axonal loss. The increased ADC is a sign of cellular injury, and the elevated AD and RD indicate axonotmesis and myelin degradation, respectively (Mailhard et al., 2013). Notably, a higher rFA but lower rADC, rAD, and rRD were detected in corresponding regions after TMP treatment, suggesting TMP could lessen microstructural injury of axons and myelin sheath and facilitate axonal remodeling after ischemic stroke. In particular, DTT maps revealed that TMP intervention robustly elevated the fiber density and length in the ischemic external capsule and internal capsule, suggesting fiber tract repair. With the information obtained from MRI, this study firmly demonstrated the efficacy of TMP in facilitating white matter reformation involving demyelination and axonal reorganization, following ischemia. However, the intricate mechanism remains unclear and requires further investigation.

Previous studies have revealed that TMP could inhibit platelet aggregation, reduce blood–brain barrier permeability, and promote angiogenesis (Tan et al., 2015; Li L et al., 2019), which were essential for maintaining circulatory homeostasis following ischemia. CBF improvement is favorable for relieving axonal injury and promoting white matter remodeling after ischemia (Hatakeyama et al., 2020). To show the association between CBF and axonal remodeling, we quantitatively investigated the CBF with MRI-ASL images and constructed the correlation between CBF and DTI indices. Our findings demonstrated that MCAO rats treated with TMP showed restoration of the CBF in peri-infarct regions. Interestingly, a strong correlation was observed between rCBF and rFA/rADC, suggesting elevating regional CBF might coordinate axonal remodeling.

On the grounds of the aforementioned results, we further investigated the level of axonal growth protein GAP-43 and synaptogenesis marker SYN. GAP-43 exits in the growth-cone membranes of axons, and SYN lies on the membranes of presynaptic vesicles. The upregulated GAP-43 and SYN are, respectively, related to neurite outgrowth and synaptic plasticity (Chung et al., 2020; Jing et al., 2020). Our results found TMP treatment markedly improved GAP-43 and SYN expressions in the peri-ischemic cortex. Furthermore, TEM images provided direct evidence that TMP could protect the ultrastructure of axons and synapses. The increased G-ratio of axons in MCAO rats treated with TMP reflected that TMP alleviated axonal damage and demyelination (Stikov et al., 2015). In particular, the synaptic ultrastructural analysis revealed that TMP induced structural changes in synaptic junctions. Synapses have dynamic structures, and the ultrastructural alterations are closely related to synaptic plasticity (Li et al., 2016). In addition, TMP could alleviate mitochondrial damage in axons and synapses. Collectively, these data combined with MRI evidence strongly support that improved CBF after TMP treatment is beneficial for axonal outgrowth and synaptic plasticity.

Recently, attractive and repulsive axonal guidance cues such as Netrin-1 and Slit-2 have been recognized to play a major role in guiding axonal growth. In this study, we found TMP could upregulate Netrin-1/DCC and Slit-2/Robo-1 levels after ischemia for 15 days. It was worth noting that TMP (40 mg/kg) significantly increased the Netrin-1 expression compared with the model group. Netrin-1 is initially characterized as a neural guidance factor and has been demonstrated as a potent vascular mitogen that stimulates proliferation, migration, and tube formation (Park et al., 2004). Subsequently, evidence revealed that Netrin-1 could induce angiogenesis and improve the post-stroke neurovascular structure in adult mouse brains (Lu et al., 2012; Ding et al., 2014). These results, along with the information obtained from ASL and DTI, raised the interesting possibility of the beneficial effects of TMP toward CBF augmentation and axonal repair after ischemic stroke.

It is evident that the growth-inhibitory proteins of myelin-associated axons make a critical difference in impeding axonal repair post stroke (Huang et al., 2017). Specifically, NogoA binding to NgR inhibits axonal sprouting by activating the downstream RhoA and its effector ROCK-2, disintegrating axonal growth cones (Zagrebelsky and Korte, 2014). More obviously, TMP-treated rats exhibited significantly reduced growth-inhibitory proteins NogoA/NgR and RhoA/ROCK-2. Our findings were consistent with previous reports showing upregulated NogoA/NgR and RhoA/ROCK-2, following ischemic stroke (Kilic et al., 2010). Overall, our findings proved that axonal guidance and growth-inhibitory signals within the cerebral microenvironment might contribute to TMP promoting brain tissue remodeling post ischemia.

Remodeling of gray and white matter accounts for functional recovery (Rosano et al., 2008; de Laat et al., 2011); thus, we investigated the therapeutic effects of TMP on gait impairment using the DigiGait-assisted automated analysis system. Unilateral ischemia could result in bilateral gait variations (Parkkinen et al., 2013), and clinical studies based on MRI have confirmed that demyelination and axonal degeneration are parallel to the attenuated gait speed, stride length, and double support time [56, 57]. The increase in steps and decline in the stride length are related to the attenuation of gait stability and speed (Krasovsky et al., 2012; van der Holst et al., 2018); in addition , the reduction in paw area is due to the inadequate propulsion and weight-bearing capabilities of the limbs (Zeng et al., 2018). In particular, the shortened hindlimb shared stance time and increased ataxia coefficient suggest that limb coordination is impaired by stroke (Langhorne et al., 2011; Ambrosini et al., 2020). In the present study, gait parameters were effectively improved after TMP treatment for 2 weeks, including steps, cadence, stride length, hindlimb shared stance time, paw area, and ataxia coefficient, demonstrating that TMP has the potential to alleviate the gait deficit of MCAO rats.

In the present research, we proved TMP alleviated gray and white matter injury and enhanced axonal remodeling by improving CBF, inducing endogenous GAP-43 and SYN expressions, augmenting guidance cues Netrin-1/DCC and Slit-2/Robo-1, and interfering with intrinsic growth-inhibitory signals NogoA/NgR and RhoA/ROCK-2. These data provide meaningful evidence that TMP might improve the intracerebral microenvironment of ischemic areas and benefit white matter remodeling, in consequence, contributing to the improvement of functional recovery after stroke.

## Data Availability

The original contributions presented in the study are included in the article/Supplementary Material; further inquiries can be directed to the corresponding author.
