# Supplementary figures and images for: Magnetic Resonance Imaging Investigation of Neuroplasticity After Ischemic Stroke in Tetramethylpyrazine-Treated Rats
Source: Front Pharmacol. 2022 Apr 26;13:851746. doi: 10.3389/fphar.2022.851746 (PMC9086494; doi:10.3389/fphar.2022.851746)

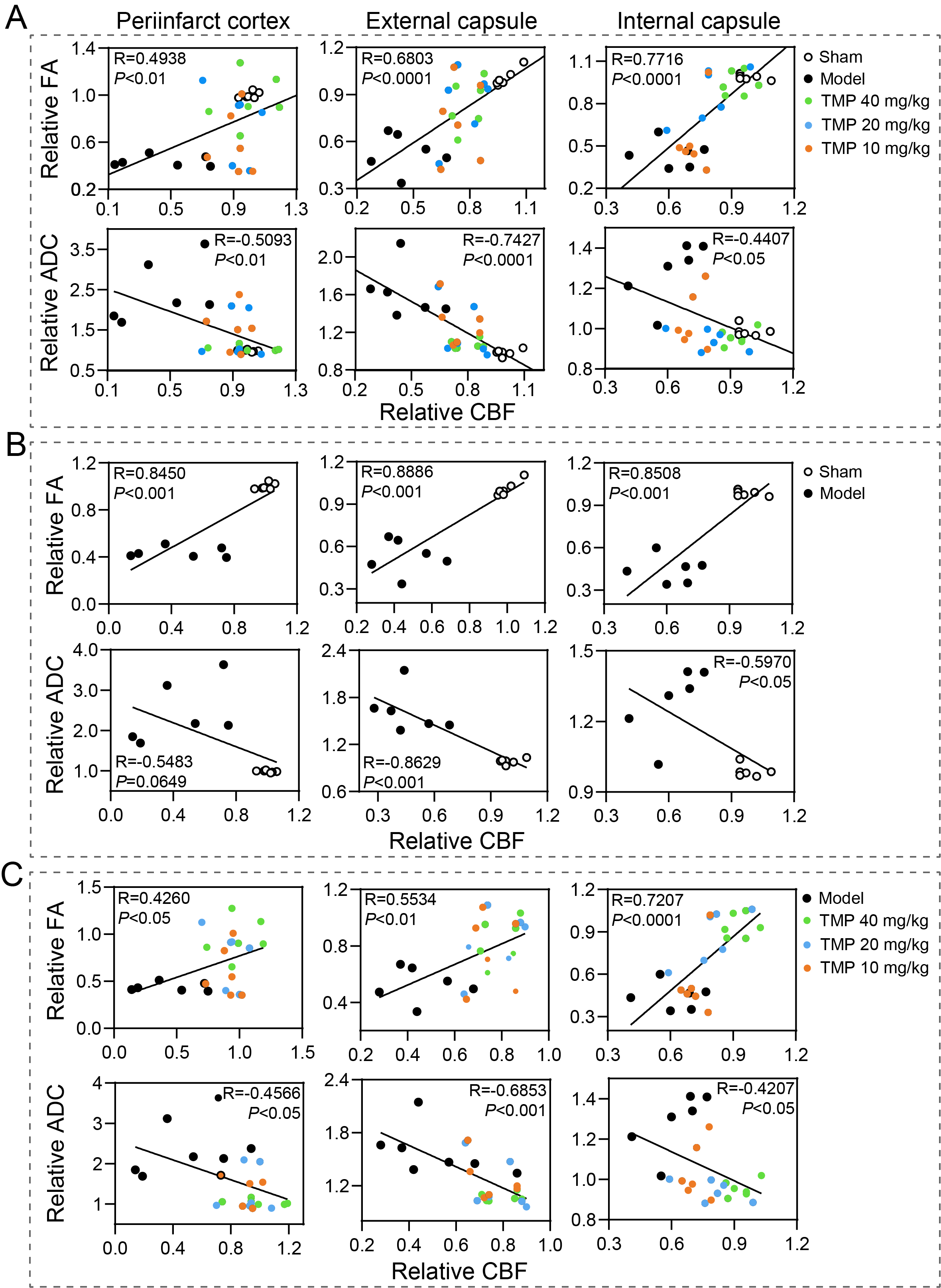

Supplement: Supplementary file 1 [file Image3.TIF]

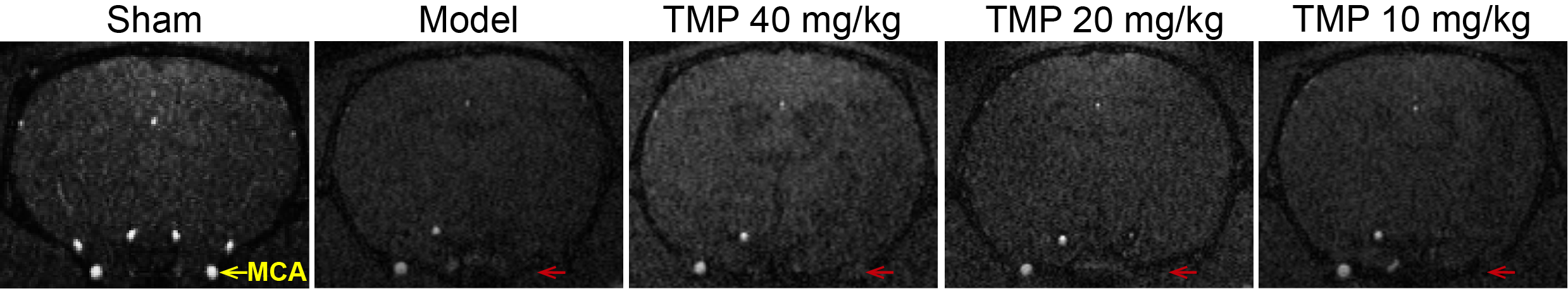

Supplement: Supplementary file 2 [file Image2.TIF]

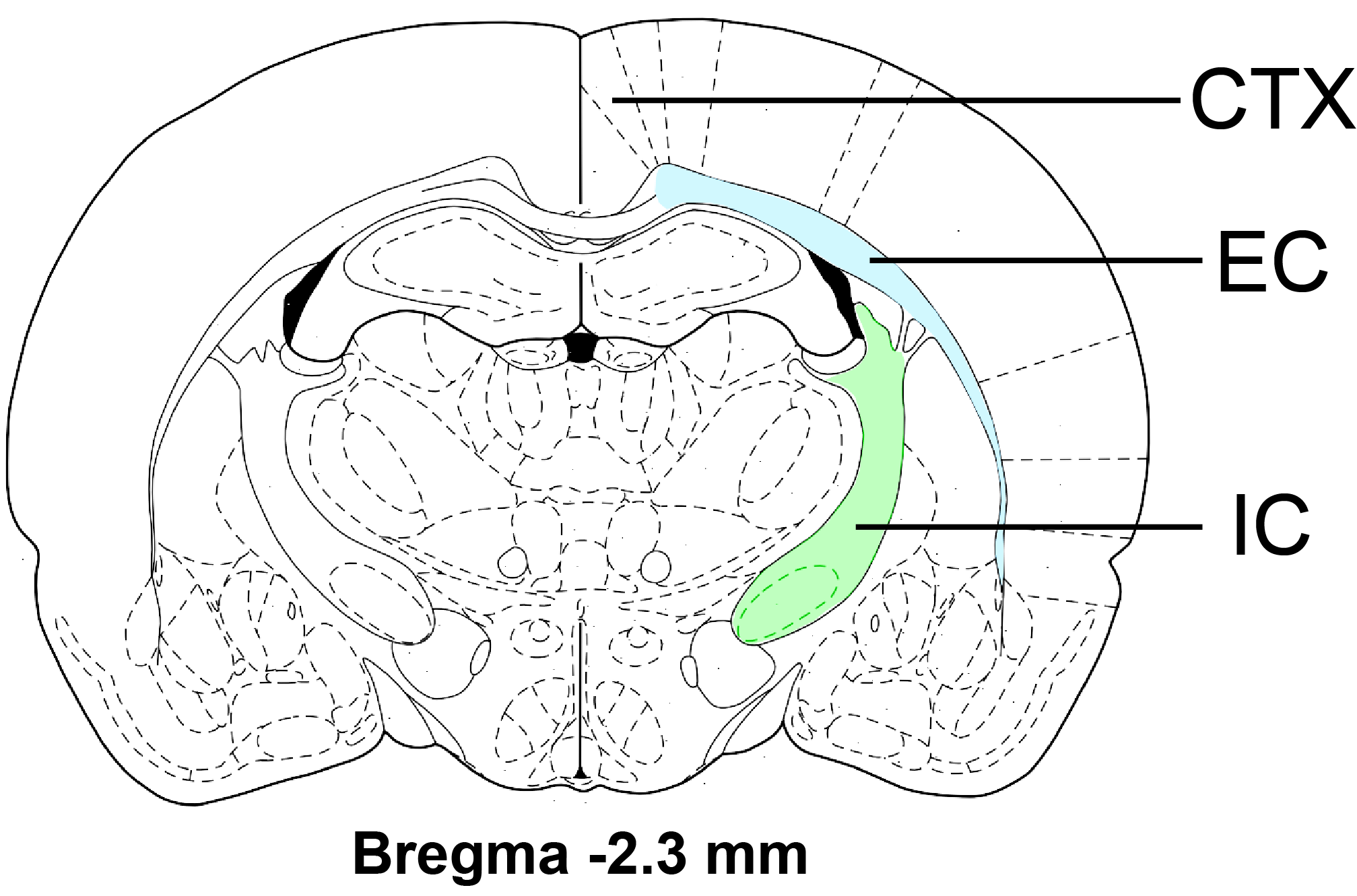

Supplement: Supplementary file 3 [file Image1.TIF]
