# Supplementary material for: Magnetic Resonance Imaging Investigation of Neuroplasticity After Ischemic Stroke in Tetramethylpyrazine-Treated Rats
Source: Front Pharmacol. 2022 Apr 26;13:851746. doi: 10.3389/fphar.2022.851746 (PMC9086494; doi:10.3389/fphar.2022.851746)
Supplement: Supplementary file 4 [file Image1.pdf]

Supplementary Figure

No.	Proteins	Western blotting images
1	GAP-43	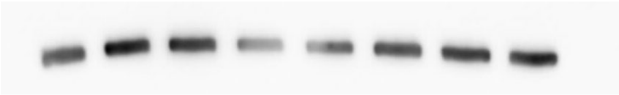
	GAPDH	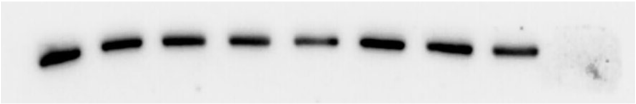
2	SYN	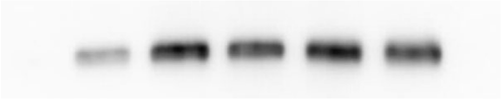
	GAPDH	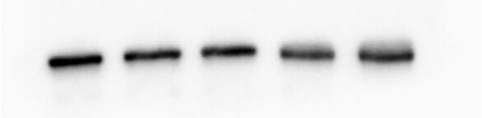
3	Netrin-1	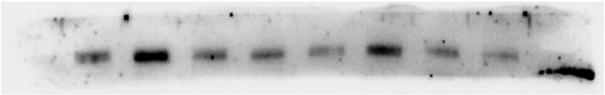
	GAPDH	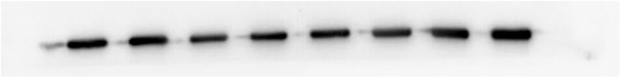
4	DCC	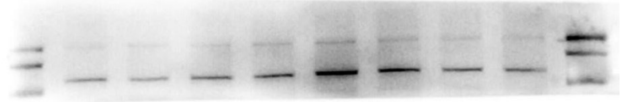
	GAPDH	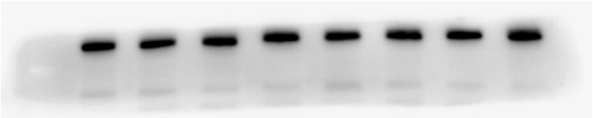
5	Slit-2	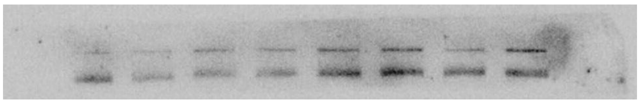
	GAPDH	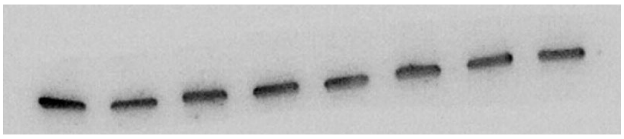
6	Robo-1	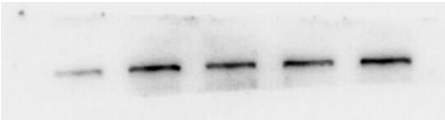
	GAPDH	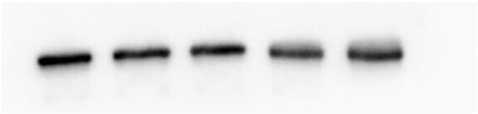
7	NogoA	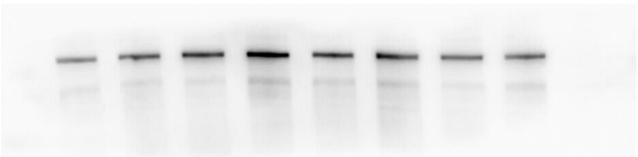

	GAPDH	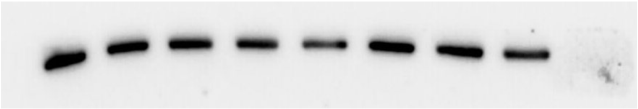
8	NgR	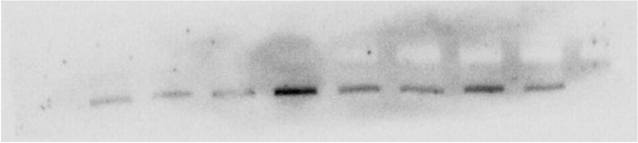
	GAPDH	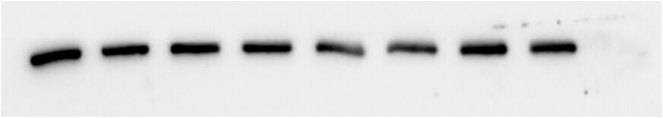
9	RhoA	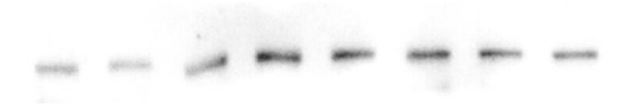
	GAPDH	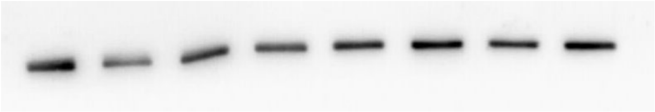
10	ROCK-2	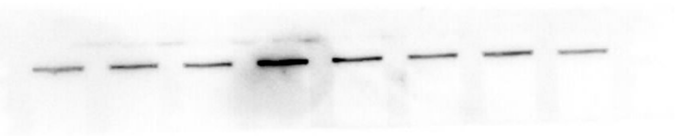
	GAPDH	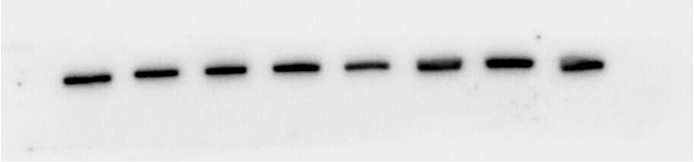
